# The pesticide dieldrin disrupts proteins related to oxidative respiration and mitochondrial stress in the central nervous system

**DOI:** 10.1016/j.dib.2017.03.008

**Published:** 2017-03-11

**Authors:** Andrew M. Cowie, Kathleena I. Sarty, Angella Mercer, Jin Koh, Karen A. Kidd, Christopher J. Martyniuk

**Affiliations:** a Canadian Rivers Institute and Department of Biology, University of New Brunswick, Saint John, New Brunswick E2L 4L5, Canada; b Interdisciplinary Center for Biotechnology Research, University of Florida, Gainesville, Florida 32611, USA

## Abstract

Quantitative proteins analysis was carried out in the hypothalamus of zebrafish following dietary exposure to the legacy pesticide dieldrin. Data were collected using iTRAQ labeling methodology and data were acquired using a hybrid quadrupole Orbitrap (Q Exactive) MS system (Thermo Fisher Scientific, Bremen, Germany). There were 3941 proteins identified in the hypothalamus of zebrafish, and these proteins comprised 23 unique expression patterns for proteins based on the three doses of dieldrin. There were 226 proteins that were regulated in one or more doses of dieldrin and 3715 proteins that were not affected. Thus, 5.7% of the proteins detected responded to the treatment. Many proteins that were differentially expressed were those found in, or associated with, the mitochondria. The proteomics data described in this article is associated with a research article, “Transcriptomic and proteomic analysis implicates the immune system and mitochondria as molecular targets of dieldrin in the zebrafish (*Danio rerio*) central nervous system” (A.M. Cowie, K.I. Sarty, A. Mercer, J. Koh, K.A. Kidd, C.J. Martyniuk, submitted) [1], and serves as a resource for researchers working in the field of pesticide exposures and protein biomarkers.

**Specifications Table**

**Table:** 

Subject area	*Biology*
More specific subject area	*Pesticide neurotoxicity, proteomics, biomarkers, pathway analysis, zebrafish*
Type of data	*Table, figure, and supplemental file of protein data in excel*
How data was acquired	Mass spectrometry using a hybrid quadrupole Orbitrap (Q Exactive) MS system (Thermo Fisher Scientific, Bremen, Germany).
Data format	*Filtered and analyzed*
Experimental factors	*Samples were subjected to SCX fractionation prior to analysis*
Experimental features	*Female adult zebrafish, 5 months of age were fed dieldrin. There were four treatment groups that included a control, and three doses of dieldrin spiked feed (0.03, 0.15, and 1.8* *µg DLD /g d.w. feed). Fish were fed for 21 days.*
Data source location	*Not applicable.*
Data accessibility	*Data are with this article.*

**Value of the data**

• Characterizes dieldrin-regulated pesticides in the zebrafish central nervous system.
• Identification of biomarker candidates for pesticide induced neurotoxicity that can be tested in further studies.
• Offers a proteomic framework for the relationship between chemical exposures and neurodegenerative diseases.
• Discovery and identification of mitochondrial proteins that are responsive to the organochlorine pesticide dieldrin and which can be compared to other studies investigating pesticide-induced mitochondrial dysfunction.

## 1 Data

Quantitative proteomics using isobaric tagging for relative and absolute quantitation (ITRAQ) was conducted in the hypothalamus of zebrafish fed the organochloride pesticide dieldrin (Cowie et al., submitted) [1]. Data were collected using iTRAQ labeling methodology and data were acquired using a hybrid quadrupole Orbitrap (Q Exactive) MS system (Thermo Fisher Scientific, Bremen, Germany). There were 3941 proteins identified in the hypothalamus of zebrafish and 226 proteins that were regulated in one or more doses of dieldrin (Supplemental Data 1). These proteins comprised 23 unique expression patterns for proteins based on the three doses of dieldrin (Table 1). The remaining proteins that were not changed (3715) are indicated in group X1.The most dramatic proteomic response in terms of protein number occurred with the high feed dose, and 61 proteins were significantly decreased (Cluster XII) with 1.8 µg DLD/g d.w. feed. Proteins in this group were not changed with the two lower doses of dieldrin. The group that contained the second highest number of proteins (*n*=27) was Expression Pattern IV, and proteins in this group were increased in all three doses of dieldrin (Table 1). Expression pattern X contained proteins that were increased in the high dieldrin treatment only (*n*=26). Approximately 5.7% of the proteins detected in this study were responsive to the dietary treatment.

**Fig. 1:**
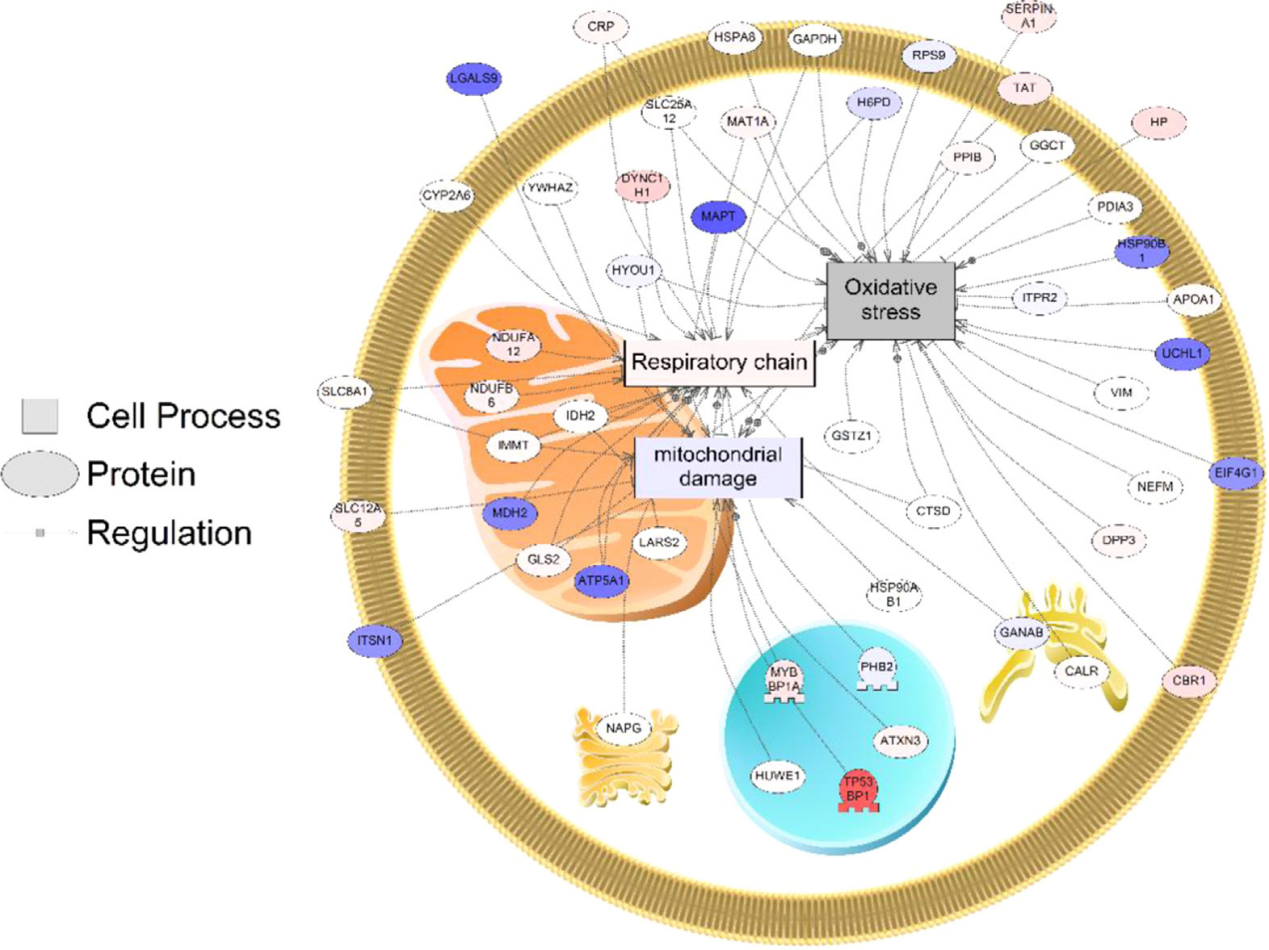
Protein network for cell processes related to mitochondrial respiration. Red indicates that protein levels are significantly increased for that protein and blue indicates that the protein levels are decreased. The more intense the color, the larger the relative fold change of the protein compared to the control group. Abbreviations are provided in Appendix 2. (For interpretation of the references to color in this figure legend, the reader is referred to the web version of this article.).

In short, the subnetwork enrichment analysis for all proteins affected by dieldrin (in one or more treatment groups) revealed that cell processes related to protein folding, actin organization, cell death, apoptosis ER unfolded protein response, protein degradation, response to drug, and neuronal death were impacted at the protein level. In other words, proteins that were significantly modulated by dieldrin were involved in these biological processes (Supplemental Data 2). In addition, proteins related to mitochondrial and oxidative phosphorylation were preferentially targeted (Supplemental Data 2, Fig. 1). Eighteen proteins involved in the respiratory chain were affected by dieldrin, for example SLC8A1 (Solute Carrier Family 8 Member A1), SLC25A12 (Solute Carrier Family 25 Member 12), IDH2 (Isocitrate Dehydrogenase (NADP(+)) 2, Mitochondrial), MDH2 (Malate Dehydrogenase 2), LARS2 (Leucyl-TRNA Synthetase 2, Mitochondrial), NAPG (NSF Attachment Protein Gamma), Mitochondrial membrane ATP synthase (F1F0 ATP synthase or Complex V), and CYP2A6 among others.

## 2 Experimental design, materials and methods

### 2.1 Protein extraction and iTRAQ labeling

Proteins following quantification were dissolved in denaturant buffer (0.1% SDS (w/v)) and dissolution buffer (0.5 M triethylammonium bicarbonate, pH 8.5) in the iTRAQ Reagents 8-plex kit (AB sciex Inc., Foster City, CA, USA). For each sample, 60 μg of protein were reduced, alkylated, trypsin-digested, and labeled according to the manufacturer׳s instructions (AB Sciex Inc.). The hypothalami were labeled with one of the iTRAQ tags (113–116) (control was labeled with 113 and the three treatments were labeled as low = 114, medium = 115, and high dose = 116). This was done for three independent biological replicates/group. Thus, there were 3 iTRAQ experiments conducted.

### 2.2 LC-MS/MS analysis

Labeled peptides were desalted with C18-solid phase extraction and dissolved in strong cation exchange (SCX) solvent A (25% (v/v) acetonitrile, 10 mM ammonium formate, and 0.1% (v/v) formic acid, pH 2.8). The peptides were fractionated using an Agilent HPLC 1260 with a polysulfoethyl A column (2.1×100 mm, 5 µm, 300 Å PolyLC, Columbia, MD, USA). Peptides were eluted with a linear gradient of 0–20% solvent B (25% (v/v) acetonitrile and 500 mM ammonium formate, pH 6.8) over 50 min., followed by ramping up to 100% solvent B in 5 min. The absorbance at 280 nm was monitored and a total of 14 fractions were collected. The fractions were lyophilized and resuspended in LC solvent A (0.1% formic acid in 97% water (v/v), 3% acetonitrile (v/v)). A hybrid quadrupole Orbitrap (Q Exactive) MS system (Thermo Fisher Scientific, Bremen, Germany) was used with high energy collision dissociation (HCD) in each MS and MS/MS cycle. The MS system was interfaced with an automated Easy-nLC 1000 system (Thermo Fisher Scientific, Bremen, Germany). Each sample fraction was loaded onto an Acclaim Pepmap 100 pre-column (20 mm × 75 μm; 3 μm-C18) and separated on a PepMap RSLC analytical column (250 mm×75 μm; 2 μm-C18) at a flow rate at 350 nl/min during a linear gradient from solvent A (0.1% formic acid (v/v)) to 25% solvent B (0.1% formic acid (v/v) and 99.9% acetonitrile (v/v)) for 80 min, and to 100% solvent B for additional 15 min.

The raw MS/MS data files were processed by a thorough database searching approach considering biological modification and amino acid substitution against the National Center for Biotechnology Information (NCBI) Teleostei database (downloaded on Mar. 25. 2015; 729,330 entries) using the ProteinPilot v4.5 with the Fraglet and Taglet searches under ParagonTM algorithm [2]. The following parameters were considered for all the searching: fixed modification of methylmethane thiosulfonate-labeled cysteine, fixed iTRAQ modification of amine groups in the N-terminus, lysine, and variable iTRAQ modifications of tyrosine. For protein quantification, only MS/MS spectra that were unique to a particular protein and where the sum of the signal-to-noise ratios for all the peak pairs > 9 were used for quantification. The accuracy of each protein ratio is given by a calculated error factor from the ProGroup analysis in the software, and a *P* value is given to assess whether the protein is significantly differentially expressed. The error factor is calculated with 95% confidence error, where it is the weighted standard deviation of the weighted average of log ratios multiplied by Student׳s *t* factor. The *P* value is determined by calculating Student׳s *t* factor by dividing the (weighted average of log ratios – log bias) by the weighted standard deviation, allowing the determination of the *P* value with *n*) 1 degrees of freedom, where *n* is the number of peptides contributing to the protein relative quantification(software default settings, AB Sciex, Inc.). To be identified as being significantly differentially expressed, a protein had to contain at least three spectra (allowing the generation of a *P* value), with *P*<0.05. Additivity of protein expression was assessed quantitatively; we calculated additive expression based on mid-parent values (MPVs; averaged values from two biological replicates of each parent). To be identified as being significantly differentially expressed, a protein was quantified with at least three unique spectra in at least two of the biological replicates, along with a Fisher׳s combined probability of <0.05 and a fold change of ±1.2.

### 2.3 Protein network analysis

For proteomics network analysis, 208 proteins that were differentially expressed and successfully mapped to Pathway Studio (Elsevier) using Name + Alias (i.e. had mammalian homologs for the zebrafish protein identified). Proteins that showed a significant changes in one or more of the doses were used to build networks, and an average fold change for the protein was obtained across each dose in which the protein was significantly different from control (*P*<0.05) (i.e., in cases where the protein was differentially expressed in multiple treatments). In 14 cases, the protein showed opposite responses in the HYP (i.e., increasing in the HYP with one dose and decreasing in the HYP following another dose). In these cases, the highest magnitude of response was used to map protein networks. The enrichment p-value for all queries was set at *P*<0.05. Subnetwork enrichment analysis was conducted in Pathway Studio for the list of proteins, and cell process was queried. These protein networks represent those that are significantly represented by proteins that are differentially regulated by dieldrin. All abbreviations for the network are provided in Supplemental Data 3.

## Figures and Tables

**Table 1:** Expression patterns in the hypothalamus compared to control group (>1.2 or <0.8 with *P*<0.05). There were 27 proteins that were increased in all three doses and one protein down-regulated in abundance in all three doses (bold).

***Expression Pattern***	***Low Dose***	***Medium Dose***	***High Dose***	***Number of Proteins***
A				3715
B			DOWN	61
C	**UP**	**UP**	**UP**	**27**
D			UP	26
E		UP		21
F	UP			14
G	UP	UP		13
H		DOWN		12
I	DOWN			11
J		UP	UP	8
K	UP		UP	6
L	DOWN		DOWN	6
M		DOWN	DOWN	5
N	UP		DOWN	4
O		UP	DOWN	3
P		DOWN	UP	2
Q	UP	UP	DOWN	1
R	DOWN		UP	1
S	DOWN	DOWN	UP	1
T	DOWN	DOWN		1
U	**DOWN**	**DOWN**	**DOWN**	**1**
V	DOWN	UP	DOWN	1
W	DOWN	UP		1
**TOTAL**				**3941**
